# Supplementary material for: Association between oral microbiome and seven types of cancers in East Asian population: a two-sample Mendelian randomization analysis
Source: Front Mol Biosci. 2023 Nov 21;10:1327893. doi: 10.3389/fmolb.2023.1327893 (PMC10702768; doi:10.3389/fmolb.2023.1327893)
Supplement: Supplementary file 1 [file DataSheet1.ZIP › Supplementary Materials/MR plots for tongue/tongue═╝/Ovarian cancer/pheno.199_to_ovarian cancer_forest.pdf]

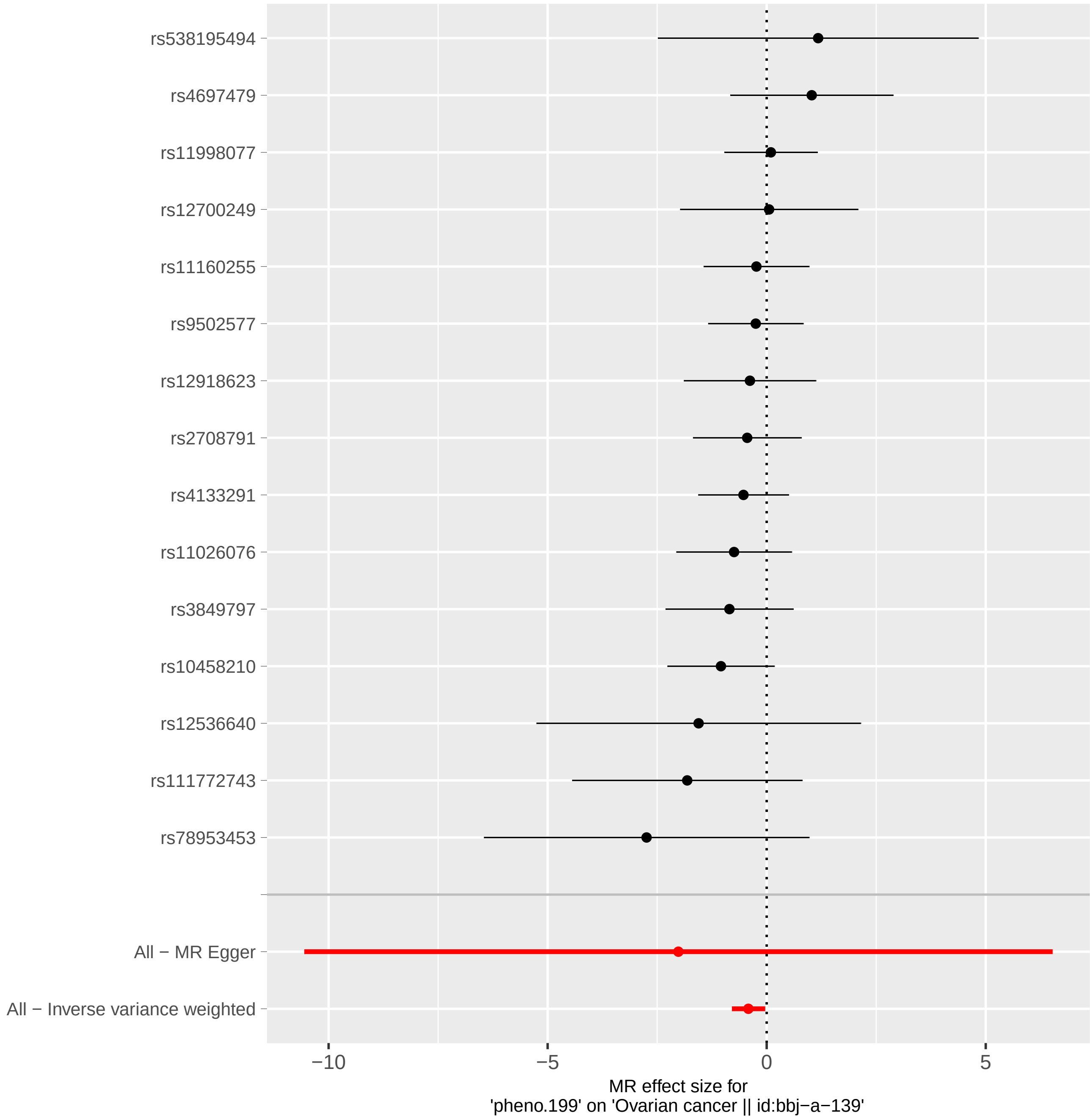
rs538195494
rs4697479
rs11998077
rs12700249
rs11160255
rs9502577
rs12918623
rs2708791
rs4133291
rs11026076
rs3849797
rs10458210
rs12536640
rs111772743
rs78953453
All − MR Egger
All − Inverse variance weighted
−10
−5
0
5
MR effect size for
'pheno.199' on 'Ovarian cancer || id:bbj−a−139'
